# Dichloridobis[2-(2-chloro­ethyl)-1,2,3,4-tetra­hydro­pyrazino[1,2-*a*]benzimidazole-κ*N*]cobalt(II)

**DOI:** 10.1107/S1600536808018011

**Published:** 2008-06-19

**Authors:** Zhong Zhang, Yao Zhao, Zhi-Rong Geng, Zhi-Lin Wang

**Affiliations:** a Coordination Chemistry Institute, State Key Laboratory of Coordination Chemistry, Nanjing University, Nanjing 210093, People’s Republic of China

## Abstract

In the title compound, [CoCl_2_(C_12_H_14_ClN_3_)_2_], the central Co^II^ ion lies on a twofold rotation axis and adopts a distorted tetra­hedral coordination geometry defined by two N atoms from two 2-(2-chloro­ethyl)-1,2,3,4-tetra­hydro­pyrazino[1,2-*a*]benzimidazole ligands and two chloride anions. The Cl atom located in the side chain of the ligand is involved in inter­molecular C—H⋯Cl hydrogen bonding, which links neutral complex units into a one-dimensional right-handed helical chain running along a crystallographic 4_1_ axis. Such hydrogen-bonded helical chains are connected to each other to form a homochiral three-dimensional supra­molecular network. One C atom of the 2-chloro­ethyl chain is disordered over two positions, with site-occupancy factors of 0.52 and 0.48.

## Related literature

For related literature, see: Balamurugan *et al.* (2004 ▶); Matrick & Day (1961 ▶); Parker *et al.* (2004 ▶); Sundberg *et al.* (1977 ▶).
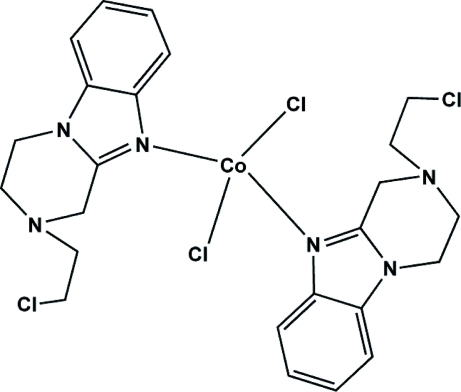


## Experimental

### 

#### Crystal data

[CoCl_2_(C_12_H_14_ClN_3_)_2_]
*M* *_r_* = 601.25
Tetragonal, 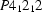

*a* = 9.5706 (8) Å
*c* = 29.911 (4) Å
*V* = 2739.7 (5) Å^3^
*Z* = 4
Mo *K*α radiation
μ = 1.04 mm^−1^
*T* = 293 (2) K
0.32 × 0.21 × 0.18 mm

#### Data collection

Bruker SMART APEX CCD area-detector diffractometer
Absorption correction: multi-scan (*SADABS*; Bruker, 2000 ▶) *T*_min_ = 0.763, *T*_max_ = 0.829
14573 measured reflections
2703 independent reflections
2219 reflections with *I* > 2σ(*I*)
*R*_int_ = 0.061

#### Refinement

*R*[*F* ^2^ > 2σ(*F* ^2^)] = 0.055
*wR*(*F* ^2^) = 0.131
*S* = 1.01
2703 reflections
169 parameters
2 restraints
H-atom parameters constrained
Δρ_max_ = 0.54 e Å^−3^
Δρ_min_ = −0.80 e Å^−3^
Absolute structure: Flack (1983 ▶), 1274 Friedel pairs
Flack parameter: 0.07 (5)

### 

Data collection: *SMART* (Bruker, 2000 ▶); cell refinement: *SAINT* (Bruker, 2000 ▶); data reduction: *SAINT*; program(s) used to solve structure: *SHELXTL* (Sheldrick, 2008 ▶); program(s) used to refine structure: *SHELXTL*; molecular graphics: *DIAMOND* (Brandenburg, 1998 ▶) and *ORTEP-3* (Farrugia, 1997 ▶); software used to prepare material for publication: *SHELXTL*.

## Supplementary Material

Crystal structure: contains datablocks I, global. DOI: 10.1107/S1600536808018011/om2235sup1.cif

Structure factors: contains datablocks I. DOI: 10.1107/S1600536808018011/om2235Isup2.hkl

Additional supplementary materials:  crystallographic information; 3D view; checkCIF report

## Figures and Tables

**Table:** 

Co1—N2	2.026 (4)
Co1—Cl2	2.2423 (13)

**Table:** 

N2^i^—Co1—N2	103.8 (2)
N2—Co1—Cl2	109.08 (12)
Cl2—Co1—Cl2^i^	110.64 (7)

Symmetry code: (i) 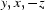.

**Table 2:** Hydrogen-bond geometry (Å, °)

*D*—H⋯*A*	*D*—H	H⋯*A*	*D*⋯*A*	*D*—H⋯*A*
C1—H1*A*⋯Cl1^ii^	0.97	2.87	3.812 (5)	164
C5—H5⋯Cl2^iii^	0.93	2.79	3.709 (5)	171

Symmetry codes: (ii) 
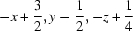
; (iii) 
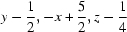
.

**Fig. 1.**
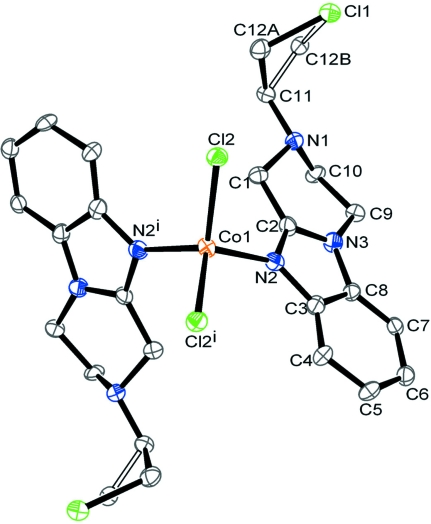
The molecular structure with atom labels and 30% probability displacement ellipsoids for non-H atoms. Both disordered components of the 2-chloroethyl chain are shown. [Symmetry code: (i) y, x, -z]

**Fig. 2.**
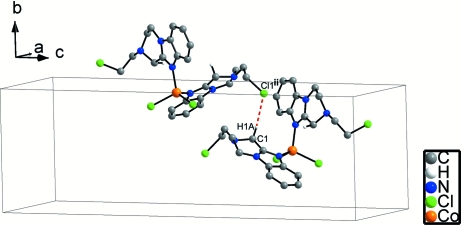
Fragment of a one-dimensional right-handed helical chain with the intermolecular C—H···Cl hydrogen bond indicated by dashed line. For clarity only the major disordered component is shown. [Symmetry code: (ii) 3/2 - x, -1/2 + y, 1/4 - z]

## supplementary crystallographic information

### Comment

Nitrogen mustards that contain a reactive *N,N*-bis-(2-chloroethyl)amine group are widely used as alkylating agents in cancer chemotherapy. However, these nitrogen mustards exhibit high chemical reactivity and usually show no selectivity of DNA alkylation. Recently, it has been proved that complexation of macrocyclic nitrogen mustards with metals may be an effective strategy in the design of hypoxia-selective antitumor prodrugs (Parker *et al.*, 2004). As part of our work, some metal complexes of a monofunctional mustard, 2-(2-chloroethyl)-l,2,3,4-tetrahydropyrazino[1,2-*a*]benzimidazole (*L*), were prepared in order to evaluate their antitumor and antimalarial activities. Here, we report the crystal structure of a novel Co(II) mustard complex.

As depicted in Fig. 1, the complete complex molecule is generated by a twofold symmetry operation, with the Co^II^ ion located on the rotation axis. The distorted tetrahedral coordination sphere around the Co^II^ center consists of two benzimidazole N atoms from two *L* ligands and two chloride anions. The Co—N distance of 2.026 (4) Å and Co—Cl length of 2.2423 (13) Å (Table 1) are comparable to those reported in the literature (Sundberg *et al.*, 1977).

In the crystal packing, intermolecular C—H···Cl hydrogen bonding play a key role. Fig. 2 illustrates that adjacent neutral complex units are connected by C1—H1A···Cl1 interactions into a one-dimensional right-handed helical architecture running along a crystallographic 4_1_ screw axis in the *c* direction. Five complex fragments form a helix turn with a long pitch of 29.911 (4) Å. The shortest intrachain Co—Co distance is 10.072 Å. Further C—H···Cl hydrogen bond linkages (Table 2) extend such one-dimensional helical chains into a homochiral three-dimensional hydrogen-bonded network (Balamurugan *et al.*, 2004).

### Experimental

The ligand 2-(2-chloroethyl)-l,2,3,4-tetrahydropyrazino[1,2-*a*]benzimidazole was synthesized according to a literature method (Matrick & Day, 1961).

The title compound was prepared by adding a methanol solution (10 ml) of CoCl_2_.6H_2_O (1 mmol) into a methanol solution (10 ml) of 2-(2-chloroethyl)-l,2,3,4-tetrahydropyrazino[1,2-*a*]benzimidazole (2 mmol). The resulting mixture was refluxed for two hours and filtered after cooling to room temperature. Blue single crystals of the title compound suitable for X-ray diffraction analysis were obtained by slow diffusion of diethyl ether into the filtrate. Elemental analysis found: C 47.80; H 4.60; N 14.03%; calculated for C_24_H_28_Cl_4_CoN_6_: C 47.94; H 4.69; N 13.98%.

### Refinement

The 2-chloroethyl chain attached to N atom displays rotational disorder and its C12 atom was split into two positions (C12A and C12B) with site-occupancy factors of 0.52 and 0.48. A l l H atoms were placed in calculated positions and refined using a riding model, with C—H = 0.93 - 0.97 Å and with *U*_iso_(H) = 1.2 *U*_eq_(C).

### Crystal data

**Table:**

[CoCl_2_(C_12_H_14_ClN_3_)_2_]	*Z* = 4
*M**_r_* = 601.25	*F*_000_ = 1236
Tetragonal, *P*4_1_2_1_2	*D*_x_ = 1.458 Mg m^−^^3^
Hall symbol: P 4abw 2nw	Mo *K*α radiation λ = 0.71073 Å
*a* = 9.5706 (8) Å	Cell parameters from 835 reflections
*b* = 9.5706 (8) Å	θ = 2.5–16.9º
*c* = 29.911 (4) Å	µ = 1.04 mm^−^^1^
α = 90º	*T* = 293 (2) K
β = 90º	Prism, blue
γ = 90º	0.32 × 0.21 × 0.18 mm
*V* = 2739.7 (5) Å^3^	

### Data collection

**Table:**

Bruker SMART APEX CCD area-detector diffractometer	2703 independent reflections
Radiation source: sealed tube	2219 reflections with *I* > 2σ(*I*)
Monochromator: graphite	*R*_int_ = 0.061
*T* = 293(2) K	θ_max_ = 26.0º
φ and ω scans	θ_min_ = 2.2º
Absorption correction: multi-scan(SADABS; Bruker, 2000)	*h* = −11→7
*T*_min_ = 0.763, *T*_max_ = 0.829	*k* = −11→11
14573 measured reflections	*l* = −36→35

### Refinement

**Table:**

Refinement on *F*^2^	Hydrogen site location: inferred from neighbouring sites
Least-squares matrix: full	H-atom parameters constrained
*R*[*F*^2^ > 2σ(*F*^2^)] = 0.055	*w* = 1/[σ^2^(*F*_o_^2^) + (0.0877*P*)^2^ + 1.82*P*] where *P* = (*F*_o_^2^ + 2*F*_c_^2^)/3
*wR*(*F*^2^) = 0.131	(Δ/σ)_max_ < 0.001
*S* = 1.01	Δρ_max_ = 0.54 e Å^−^^3^
2703 reflections	Δρ_min_ = −0.80 e Å^−^^3^
169 parameters	Extinction correction: none
2 restraints	Absolute structure: Flack (1983), 1274 Friedel pairs
Primary atom site location: structure-invariant direct methods	Flack parameter: 0.07 (5)
Secondary atom site location: difference Fourier map	

### Special details

**Table:**

Geometry. All e.s.d.'s (except the e.s.d. in the dihedral angle between two l.s. planes) are estimated using the full covariance matrix. The cell e.s.d.'s are taken into account individually in the estimation of e.s.d.'s in distances, angles and torsion angles; correlations between e.s.d.'s in cell parameters are only used when they are defined by crystal symmetry. An approximate (isotropic) treatment of cell e.s.d.'s is used for estimating e.s.d.'s involving l.s. planes.
Refinement. Refinement of *F*^2^ against ALL reflections. The weighted *R*-factor *wR* and goodness of fit *S* are based on *F*^2^, conventional *R*-factors *R* are based on *F*, with *F* set to zero for negative *F*^2^. The threshold expression of *F*^2^ > σ(*F*^2^) is used only for calculating *R*-factors(gt) *etc*. and is not relevant to the choice of reflections for refinement. *R*-factors based on *F*^2^ are statistically about twice as large as those based on *F*, and *R*- factors based on ALL data will be even larger.

### Fractional atomic coordinates and isotropic or equivalent isotropic displacement parameters (Å^2^)

**Table:**

	*x*	*y*	*z*	*U*_iso_*/*U*_eq_	Occ. (<1)
Co1	0.99857 (7)	0.99857 (7)	0.0000	0.0305 (2)	
C1	0.7722 (5)	0.8869 (5)	0.07778 (15)	0.0312 (10)	
H1A	0.7825	0.7909	0.0679	0.037*	
H1B	0.8635	0.9211	0.0866	0.037*	
C2	0.7177 (5)	0.9724 (5)	0.04111 (16)	0.0327 (11)	
C3	0.6905 (5)	1.0950 (5)	−0.01903 (17)	0.0336 (11)	
C4	0.7080 (5)	1.1677 (5)	−0.05859 (18)	0.0332 (11)	
H4	0.7945	1.1750	−0.0726	0.040*	
C5	0.5873 (5)	1.2300 (5)	−0.07639 (16)	0.0339 (11)	
H5	0.5940	1.2775	−0.1034	0.041*	
C6	0.4595 (6)	1.2234 (5)	−0.05550 (17)	0.0368 (12)	
H6	0.3826	1.2693	−0.0675	0.044*	
C7	0.4469 (5)	1.1478 (5)	−0.01639 (16)	0.0317 (11)	
H7	0.3604	1.1398	−0.0024	0.038*	
C8	0.5617 (5)	1.0847 (5)	0.00170 (18)	0.0335 (11)	
C9	0.4822 (5)	0.9696 (5)	0.07306 (16)	0.0325 (11)	
H9A	0.3964	0.9399	0.0587	0.039*	
H9B	0.4612	1.0496	0.0918	0.039*	
C10	0.5382 (5)	0.8555 (5)	0.10051 (16)	0.0354 (12)	
H10A	0.4780	0.8397	0.1261	0.042*	
H10B	0.5418	0.7701	0.0831	0.042*	
C11	0.7325 (6)	0.8092 (5)	0.15130 (16)	0.0348 (11)	
H11A	0.7724	0.7247	0.1387	0.042*	0.520 (13)
H11B	0.6558	0.7816	0.1706	0.042*	0.520 (13)
H11C	0.8335	0.8174	0.1515	0.042*	0.480 (13)
H11D	0.7095	0.7120	0.1458	0.042*	0.480 (13)
C12A	0.8428 (10)	0.8809 (9)	0.1796 (3)	0.033 (3)	0.520 (13)
H12A	0.9132	0.9212	0.1601	0.039*	0.520 (13)
H12B	0.8883	0.8121	0.1984	0.039*	0.520 (13)
C12B	0.6908 (11)	0.8567 (10)	0.1978 (3)	0.034 (3)	0.480 (13)
H12C	0.7154	0.7845	0.2192	0.040*	0.480 (13)
H12D	0.5903	0.8691	0.1988	0.040*	0.480 (13)
N1	0.6779 (4)	0.8924 (5)	0.11560 (13)	0.0345 (9)	
N2	0.7894 (4)	1.0231 (4)	0.00683 (13)	0.0341 (9)	
N3	0.5833 (4)	1.0082 (5)	0.03979 (13)	0.0359 (9)	
Cl1	0.77153 (12)	1.01254 (13)	0.21326 (4)	0.0341 (3)	
Cl2	1.10525 (13)	1.08043 (13)	0.06139 (4)	0.0345 (3)	

### Atomic displacement parameters (Å^2^)

**Table:**

	*U*^11^	*U*^22^	*U*^33^	*U*^12^	*U*^13^	*U*^23^
Co1	0.0302 (3)	0.0302 (3)	0.0311 (4)	−0.0010 (4)	0.0031 (3)	−0.0031 (3)
C1	0.033 (3)	0.030 (2)	0.030 (2)	−0.004 (2)	−0.003 (2)	0.001 (2)
C2	0.036 (3)	0.032 (3)	0.031 (2)	0.003 (2)	0.012 (2)	0.005 (2)
C3	0.035 (3)	0.028 (3)	0.038 (3)	0.000 (2)	−0.009 (2)	−0.007 (2)
C4	0.029 (3)	0.032 (3)	0.039 (3)	0.001 (2)	−0.005 (2)	0.007 (2)
C5	0.036 (3)	0.028 (3)	0.037 (3)	−0.006 (2)	−0.002 (2)	0.017 (2)
C6	0.038 (3)	0.032 (3)	0.040 (3)	0.005 (2)	−0.002 (2)	−0.003 (2)
C7	0.032 (3)	0.032 (3)	0.031 (2)	0.005 (2)	0.000 (2)	−0.0074 (19)
C8	0.031 (3)	0.035 (3)	0.035 (2)	0.005 (2)	−0.009 (2)	−0.007 (2)
C9	0.031 (3)	0.035 (3)	0.031 (2)	−0.005 (2)	0.002 (2)	0.0002 (19)
C10	0.036 (3)	0.041 (3)	0.029 (2)	0.001 (2)	0.003 (2)	0.016 (2)
C11	0.040 (3)	0.036 (3)	0.029 (3)	0.002 (2)	−0.002 (2)	−0.003 (2)
C12A	0.036 (5)	0.031 (5)	0.031 (5)	0.000 (4)	−0.002 (4)	0.007 (4)
C12B	0.032 (6)	0.032 (6)	0.036 (6)	0.001 (4)	−0.003 (4)	0.002 (4)
N1	0.039 (2)	0.035 (2)	0.030 (2)	0.0055 (19)	0.0004 (18)	0.0035 (18)
N2	0.035 (2)	0.037 (2)	0.0303 (19)	0.0010 (18)	−0.0003 (17)	−0.0028 (17)
N3	0.036 (2)	0.035 (2)	0.037 (2)	−0.0003 (19)	0.0060 (18)	0.005 (2)
Cl1	0.0330 (6)	0.0355 (6)	0.0339 (6)	−0.0082 (5)	−0.0012 (5)	−0.0007 (5)
Cl2	0.0367 (7)	0.0333 (7)	0.0336 (6)	−0.0011 (5)	−0.0071 (5)	−0.0038 (5)

### Geometric parameters (Å, °)

**Table:**

Co1—N2^i^	2.026 (4)	C9—N3	1.436 (6)
Co1—N2	2.026 (4)	C9—C10	1.468 (6)
Co1—Cl2	2.2423 (13)	C9—H9A	0.9700
Co1—Cl2^i^	2.2423 (13)	C9—H9B	0.9700
C1—N1	1.448 (6)	C10—N1	1.454 (7)
C1—C2	1.465 (6)	C10—H10A	0.9700
C1—H1A	0.9700	C10—H10B	0.9700
C1—H1B	0.9700	C11—N1	1.431 (6)
C2—N2	1.325 (6)	C11—C12A	1.517 (10)
C2—N3	1.332 (6)	C11—C12B	1.517 (10)
C3—C4	1.383 (7)	C11—H11A	0.9700
C3—C8	1.383 (7)	C11—H11B	0.9700
C3—N2	1.403 (6)	C11—H11C	0.9700
C4—C5	1.405 (7)	C11—H11D	0.9700
C4—H4	0.9300	C12A—Cl1	1.752 (9)
C5—C6	1.374 (7)	C12A—H11C	1.0395
C5—H5	0.9300	C12A—H12A	0.9700
C6—C7	1.381 (7)	C12A—H12B	0.9700
C6—H6	0.9300	C12B—Cl1	1.742 (9)
C7—C8	1.366 (7)	C12B—H12C	0.9700
C7—H7	0.9300	C12B—H12D	0.9700
C8—N3	1.370 (7)		
			
N2^i^—Co1—N2	103.8 (2)	N1—C10—C9	109.2 (4)
N2^i^—Co1—Cl2	112.03 (12)	N1—C10—H10A	109.8
N2—Co1—Cl2	109.08 (12)	C9—C10—H10A	109.8
N2^i^—Co1—Cl2^i^	109.08 (12)	N1—C10—H10B	109.8
N2—Co1—Cl2^i^	112.03 (12)	C9—C10—H10B	109.8
Cl2—Co1—Cl2^i^	110.64 (7)	H10A—C10—H10B	108.3
N1—C1—C2	110.0 (4)	N1—C11—C12A	114.8 (5)
N1—C1—H1A	109.7	N1—C11—C12B	114.9 (5)
C2—C1—H1A	109.7	N1—C11—H11A	108.6
N1—C1—H1B	109.7	C12A—C11—H11A	108.6
C2—C1—H1B	109.7	N1—C11—H11B	108.6
H1A—C1—H1B	108.2	C12A—C11—H11B	108.6
N2—C2—N3	112.5 (4)	H11A—C11—H11B	107.6
N2—C2—C1	126.8 (5)	N1—C11—H11C	109.0
N3—C2—C1	120.6 (4)	C12B—C11—H11C	103.4
C4—C3—C8	121.8 (5)	N1—C11—H11D	109.0
C4—C3—N2	129.6 (5)	C12B—C11—H11D	112.5
C8—C3—N2	108.6 (4)	H11C—C11—H11D	107.8
C3—C4—C5	116.0 (5)	C11—C12A—Cl1	112.0 (6)
C3—C4—H4	122.0	C11—C12A—H12A	109.2
C5—C4—H4	122.0	Cl1—C12A—H12A	109.2
C6—C5—C4	122.7 (4)	C11—C12A—H12B	109.2
C6—C5—H5	118.7	Cl1—C12A—H12B	109.2
C4—C5—H5	118.7	H12A—C12A—H12B	107.9
C5—C6—C7	119.2 (5)	C11—C12B—Cl1	112.5 (6)
C5—C6—H6	120.4	C11—C12B—H12C	109.1
C7—C6—H6	120.4	Cl1—C12B—H12C	109.1
C8—C7—C6	119.8 (5)	C11—C12B—H12D	109.1
C8—C7—H7	120.1	Cl1—C12B—H12D	109.1
C6—C7—H7	120.1	H12C—C12B—H12D	107.8
C7—C8—N3	133.4 (5)	C11—N1—C1	109.6 (4)
C7—C8—C3	120.5 (5)	C11—N1—C10	115.6 (4)
N3—C8—C3	106.1 (4)	C1—N1—C10	108.8 (4)
N3—C9—C10	109.4 (4)	C2—N2—C3	104.9 (4)
N3—C9—H9A	109.8	C2—N2—Co1	123.2 (3)
C10—C9—H9A	109.8	C3—N2—Co1	131.9 (3)
N3—C9—H9B	109.8	C2—N3—C8	107.9 (4)
C10—C9—H9B	109.8	C2—N3—C9	124.4 (4)
H9A—C9—H9B	108.2	C8—N3—C9	127.7 (4)

Symmetry codes: (i) *y*, *x*, −*z*.

### Hydrogen-bond geometry (Å, °)

**Table:**

*D*—H···*A*	*D*—H	H···*A*	*D*···*A*	*D*—H···*A*
C1—H1A···Cl1^ii^	0.97	2.87	3.812 (5)	164
C5—H5···Cl2^iii^	0.93	2.79	3.709 (5)	171

Symmetry codes: (ii) −*x*+3/2, *y*−1/2, −*z*+1/4; (iii) *y*−1/2, −*x*+5/2, *z*−1/4.
